# Anais Brasileiros de Dermatologia: management experiences and perspectives of a Latin American scientific journal –2021-2025 period^؆^

**DOI:** 10.1016/j.abd.2025.501240

**Published:** 2025-11-06

**Authors:** Sílvio Alencar Marques, Ana Maria Roselino, Hiram Larangeira de Almeida, Luciana Patrícia Fernandes Abbade

**Affiliations:** a Department of Infectology, Dermatology, Diagnostic Imaging and Radiotherapy, Faculty of Medicine, Universidade Estadual Paulista, Botucatu, SP, Brazil; b Department of Internal Medicine, Division of Dermatology, Faculdade de Medicina de Ribeirão Preto, Universidade de São Paulo, Ribeirão Preto, SP, Brazil; c Department of Dermatology, Universidade Federal de Pelotas, Pelotas, SP, Brazil; d Department of Dermatology, Universidade Católica de Pelotas, Pelotas, SP, Brazil

Dear Colleagues,

After five years of working with authors, reviewers, and readers, we, the current Editors, have reached the end of our tenure at the helm of the Anais Brasileiros de Dermatologia (ABD). It is time to report and highlight the implemented changes and the challenges ahead. We understand that the dynamics of the succession process in scientific journals, and in the ABD in particular, are similar to the passing of the baton in a relay race. The principle is to do our best to hand over management in the best possible position to the new leaders, who, in turn, will do their best to hand over the baton in an even better position than when they received it. This philosophy has been clearly demonstrated and practiced over the last 25 years of the ABD's history.^1^ This change in Editors is welcome because it brings new experiences and different perspectives.

As explained in previous Editorials, particularly those in volume 99 (1)^2^ and volume 100 (4),^3^ the metrics used to analyze scientific journals may incorporate biases depending on the methodologies employed and certainly require different interpretations when comparing different medical specialties. However, despite these facts, the Editors of a scientific journal must reflect on future paths, different proposals, and initiatives to achieve a better ranking when comparing their metrics with those of similar journals. Therefore, based on the Impact Factor, CiteScore, and Scimago Journal Rank (SJR) reports, and aware that each has its own methodology and different databases, this ABD management took several initiatives, notably eliminating low-impact sections, transforming others into letters, expanding the clinical cases section, including therapy letters, and expanding space for research and review articles. This was evidently done gradually, as the existence of articles approved for a given section implied its maintenance until the articles allocated to it were exhausted. Another imperative initiative for change was the need to reduce the waiting time for publication. We did this by reducing the number of accepted articles, increasing the number of articles per issue, and accelerating the publishing of online articles, which proved effective. Furthermore, inherent to the importance of a scientific journal is its visibility and its consistency in publishing what is of greatest interest to readers. However, without neglecting the graphic quality and the quality of clinical, histological, dermoscopic, and general images. As a result of the changes implemented over the period and the collective effort of Scientific and Technical Editors, Authors, and Reviewers, the ABD expanded its scope, gained greater visibility, and improved all its metrics. For example, the impact factor grew from 2.1 in 2021 to 3.6 in 2024 and rose from the 3rd quartile to the 1st quartile among 96 audited journals.^3^ These were also determining factors for the growth of the ABD, maintaining and expanding its plural tradition: articles on infectious dermatology coexist with articles on micrographic surgery, the ones on tropical dermatology with those on cosmiatry, those on basic research with those on clinical or epidemiological research.^4^, ^5^, ^6^ Moreover, it houses articles on stomatology originating from or in association with Schools of Dentistry, on chronic ulcers with the participation of or originating from Departments and Schools of Nursing.^7^, ^8^, ^9^, ^10^

It is also consolidating itself as a scientific journal with international reach, featuring articles from several countries and, as expected, with an emphasis on those from Ibero-Latin America. Of course, there are challenges to overcome, including one that could have a growing impact on scientific journals: the correct and potentially incorrect use of Artificial Intelligence (AI). The ABD will have a page in their rules that will indicate how and when to declare the use of AI, for which and what insertions in the submitted text. However, it is already clear that the use of AI to improve text quality in terms of fluency, grammatical correctness, and English editing is permitted and even welcome. However, whether or not reviewers will allow the use of AI, among other issues inherent to scientific texts, will be a topic of internal and external debate.

Other issues of collective impact, such as whether the ABD is online-only, whether or not to charge for article publication, and if so, from everyone, including members? These issues will be debated and voted on by the Board of the Brazilian Society of Dermatology (SBD, *Sociedade Brasileira de Dermatologia*). Furthermore, how to address the apparent cultural reasons that prevent some authors publishing abroad from citing the ABD, even when pertinent articles exist and are authored by the authors themselves. Decisions have already been made and are yet to be implemented, such as having the ABD published in print and online exclusively in English, which will be challenging at first. And, certainly, new proposals and initiatives will be debated and implemented. But, as mentioned above, this involves rotating leadership, with different names or roles, but with the same foundations, objectives, and certainly new visions, methodologies, and requirements.... *E pur si muove*

Our thanks to the Authors and Reviewers who have walked with us, to the SBD Board of Directors for their unwavering support, to Elsevier Publishing for their presence and contribution to the discussions and improvements implemented, and to the Technical Editors for their excellence. And welcome and best wishes for success to the new Editors, for the 2026–2031 term.

## ORCID IDs

Sílvio Alencar Marques: 0000-0002-5512-4700; Ana Maria Roselino: 0000-0002-2709-1825; Hiram Larangeira de Almeida Junior: 0000-0003-0705-1778; Luciana Patrícia Fernandes Abbade: 0000-0002-0334-2079

## Authors' contributions

Silvio Alencar Marques: Approval of the final version of the manuscript; drafting and editing of the manuscript.

Ana Maria Roselino: Approval of the final version of the manuscript; drafting and editing of the manuscript.

Hiram Larangeira de Almeida Junior: Approval of the final version of the manuscript; drafting and editing of the manuscript.

Luciana P. Fernandes Abbade: Approval of the final version of the manuscript; drafting and editing of the manuscript.

## Research data availability

Not applicable.

## Financial support

None to declare.

## Conflicts of interest

None declared.
